# A Comparison of Complication Rates in Flexor Tendon Repair Performed in Operating Rooms Versus Clinic-Based Procedure Rooms

**DOI:** 10.1177/22925503241309926

**Published:** 2025-01-17

**Authors:** Laurent Tessier, Ariane Gélinas, Alexandra Speak, Donald Lalonde, Jacques Haddad

**Affiliations:** 1 Plastic Surgery Division, 7321 Université de Sherbrooke, Sherbrooke, Quebec, Canada

**Keywords:** WALANT, flexor tendon, flexor tendon repair, field sterility, wide awake, minor procedure room, main operating room

## Abstract

**Background:** Wide-awake local anesthesia no tourniquet (WALANT) surgery has demonstrated its value in hand surgery allowing surgeons to safely operate patients in different settings outside of a formal operating room (OR). Flexor tendon lacerations have historically been repaired in the controlled setting of an OR. Plastic surgeons at our university-affiliated center have increasingly been performing flexor repairs in clinic-based procedure rooms (PRs). This study set out to evaluate the safety and complication rates of primary flexor tendon repair performed in PRs compared to those performed in the OR. **Method:** A unicentric retrospective study was conducted with patients who underwent primary flexor tendon repair between 2019 and 2023 in both clinic-based PRs and the OR. Primary outcomes included presence of tendon rupture and secondary outcomes included infection, adhesion, reintervention, and presence of any complication. Results are reported using odds ratios with 95% confidence intervals. **Results:** One hundred seventy-four patients underwent flexor tendon repair. There was no association between rupture rate and surgical setting (OR 1.05 95% CI [0.30-3.78]; *P* = .94). Surgeries performed in clinic-based PRs showed a reduction in the odds of observing at least one complication (OR = 0.49 [0.24-0.97]; *P* = .041). A subanalysis of single digit cases showed a similar association between the rate of complications and surgical setting (OR = 0.39 [0.16-0.96]; *P* = .039). **Conclusions:** This study confirms the safety of performing flexor repair in clinic-based PRs. Such settings also offer the advantage of reduced cost of surgery and minimized delay between diagnosis and surgery.

## Résumé

**Contexte :** La chirurgie menée chez des patients pleinement éveillés sous anesthésie locale sans garrot («WALANT: Wide-Awake Local Anesthesia No Tourniquet) a montré son intérêt dans la chirurgie de la main. Elle permet aux chirurgiens d’opérer des patients sans danger dans différents cadres en dehors de celui d’une salle d’opération. Traditionnellement, les lacérations des tendons fléchisseurs ont été réparées dans un cadre contrôlé en salle d’opération. Dans notre centre affilié à une université, les chirurgiens plasticiens ont de plus en plus souvent réalisé les réparations des tendons fléchisseurs dans les salles de traitement des cliniques. Cette étude avait pour but d’évaluer l’innocuité et le taux de complications des réparations du tendon du fléchisseur commun réalisées dans des salles de procédures comparativement à celles réalisées en salles d’opération. **Méthodes :** Une étude menée dans un seul centre a inclus rétrospectivement des patients ayant subi une réparation du tendon du fléchisseur commun entre 2019 et 2023, aussi bien dans des salles de procédures en clinique qu’en salle d’opération. Les critères d’évaluation principaux étaient, notamment, l’existence d’une rupture tendineuse, et les critères d’évaluation secondaire étaient l’infection, les adhérences, une réintervention et l’existence de toute complication. Les résultats ont été rapportés en utilisant des rapports de cotes avec des intervalles de confiance de 95 %. **Résultats :** Un total de 174 patients ont subi au moins une réparation d’un tendon fléchisseur de la main. Aucune association n’a été trouvée entre le taux de rupture et le cadre chirurgical (RC: 1,05 [0,30 à 3,78]; *P *= 0,94). Les interventions pratiquées en salle de procédure en clinique avaient une réduction du risque d’observation d’au moins une complication (RC: 0,49 [0,24 à 0,97]; *P* = 0,041). Une sous-analyse des cas concernant un seul doigt a montré une association similaire entre le taux de complications et le cadre chirurgical (RC = 0,39 [0,16 à 0,96]; *P* = 0,039). **Conclusion :** Cette étude confirme qu’il est sécuritaire de réaliser une réparation des fléchisseurs dans les salles de procédures des cliniques. De tels cadres offrent également l’avantage d’une réduction des coûts de la chirurgie et du délai écoulé entre le diagnostic et l’intervention.

## Introduction

Flexor tendon repair is one of the most common pathologies of hand injury. For ideal outcomes, primary flexor tendon repair is to be performed in a timely manner after the injury^1^ and, traditionally, in the controlled setting of an operating room (OR). Major complications observed following this surgery are rupture, infection, and adhesions.^2^

Wide-awake local anesthesia no tourniquet (WALANT) surgery has demonstrated its value and popularity in hand surgery,^3^ allowing surgeons to safely operate patients in different settings, without an anesthesia team, and sometimes outside of the main OR. Many cost-analysis studies demonstrate the advantage of this practice in hand surgery.^4,5^ Flexor tendon repair using WALANT has previously been described,^6^ with good outcomes and complications profiles.^7–9^

In the wake of the COVID-19 pandemic, OR access became more limited,^10^ prompting plastic surgeons at our university to operate flexor tendons in clinic-based procedure rooms (PRs).^11^ This practice is not well described in the literature, and therefore the complication rates in such a setting are not known.^12^

We conducted a retrospective study comparing 2 different surgical settings within a single medical center to answer the following guiding question: in patients undergoing primary flexor tendon repair, does the PR setting put patients at higher risk of complications when compared to patients operated in an OR? We hypothesize that the complication rate would not be different between the 2 settings. The goal of our team was to gain a better understanding of the impact of operative setting on complications in hand flexor tendon repair.

## Methods

### Patient Sample

This retrospective study done at a single-center university hospital was approved by our institutional review board. Inclusion criteria included patients who presented with a hand flexor tendon laceration between January 1st, 2018 and February 28th, 2023. Lesions of the following structures were considered: flexor pollicis longus (FPL), flexor digitorum profundus (FDP), flexor digitorum superficialis (FDS), palmaris longus (PL), flexor carpi radialis (FCR), and flexor carpi ulnaris (FCU). Patients with concomitant major hand trauma (amputation of a digit, vascular trauma, unstable soft tissue coverage, or gross contamination) and with less than 6 months of follow up were excluded. Patients were identified via billing codes for flexor tendon repair.

Data collection was realized through retrospective chart review. Patient characteristics variables included age, gender, tobacco use, and diabetes. Trauma details collected included zone of flexor tendon injury (I through V), tendon affected and repaired, type of anesthesia, time between diagnosis and surgery, any concomitant lesions (digital nerve or artery, metacarpal or phalangeal fracture), and operative setting (OR vs outpatient clinic). Procedural type of anesthesia was described as well, namely, general, regional, or WALANT. Study groups were formed based on operative setting.

All postoperative visit notes were examined to evaluate the presence of primary and secondary outcomes. The primary outcome was defined as presence of postoperative tendon rupture, which was identified by mention of tendon rupture at follow up and/or presence of secondary tendon repair surgery. Secondary outcomes included presence of infection (surgical site infection, abscess, or flexor tenosynovitis), presence of adhesions, need to reoperate (primary flexor tendon repair, secondary tendon reconstruction, or tenolysis) and, more broadly, presence of any complication. Antibiotic prescription at follow up was considered a proxy for infectious complication. Mention of adhesions in follow-up notes, or if flexor tenolysis was offered to a patient, were considered proxies for adhesions. All files were examined up to 1 year postinjury.

Postoperative care was done in collaboration with our occupational therapy team, with an early active motion protocol given to all patients. There was no distinction in the rehabilitation offered and received by patients on the basis of whether they were operated in a clinic-based PR or in OR.

### Statistical Analysis

For comparison of study groups, chi-square (X^2^) and Mann–Whitney U tests were done for categorical variables and continuous variables, respectively. A logistic regression model was then employed to determine the association between complication rate and surgical setting. Results are reported with ORs and 95% confidence intervals. A *P*-value of less than .05 was considered significant.

## Results

After chart review, a total of 174 patients were included in the study, with 93 patients in the PR group and 81 patients in the main OR group. All demographic data collected can be found in Table 1. The study population was comprised of 137 male patients and 37 female patients. The median age was 41 years.

**Table 1.** Demographic Data.

		Outpatient clinic (*n* = 93)	Operating room (*n* = 81)	Total (*n* = 174)	*P*-value
Age at time of surgery (years)		42.5 (12-76)	42.1 (7-76)	42.3 (7-76)	.90
Sex					.30
	Female	17 (18.28%)	20 (24.69%)	37 (21.26%)	
	Male	76 (81.72%)	61 (75.31%)	137 (78.74%)	
Comorbidities					
	Tobacco use	20 (21.51%)	22 (27.16%)	42 (24.14%)	.87
	Diabetes mellitus	3 (3.23%)	6 (7.41%)	9 (5.17%)	.32

Table 2 summarizes the surgical characteristics of the 2 study groups. As for the anatomical distribution of the injuries, 58 (33.3%) patients had a lesion in flexor zone I, 73 (42.0%) in zone II, 13 (7.5%) in zone III, 2 (1.1%) in zone IV, and 28 (16.1%) in zone V. A total of 324 flexor tendon repairs were performed among the 174 patients (159 FDP, 91 FDS, 31 FPL, 16 FCR, 16 FCU, and 10 PL).

**Table 2.** Surgical Data.

		Outpatient clinic (*n* = 93)	Operating room (*n* = 81)	Total (*n* = 174)	*P*-value
Time between announcement and procedure (mins)		117 (0-2709)	613 (53-3284)	343 (0-3284)	
Laceration zone					
	Zone I	41 (44.09%)	17 (20.99%)	58 (33.33%)	
	Zone II	35 (37.63%)	38 (46.91%)	73 (41.95%)	
	Zone III	6 (6.45%)	7 (8.64%)	13 (7.47%)	
	Zone IV	1 (1.08%)	1 (1.23%)	2 (1.15%)	
	Zone V	10 (10.75%)	18 (22.22%)	28 (16.09%)	
Anesthesia					<.001
	WALANT	93 (100%)	9 (11.11%)	102 (58.62%)	
	Locoregional anesthesia	0 (0%)	47 (58.02%)	47 (27.01%)	
	General anesthesia	0 (0%)	25 (30.86%)	25 (14.37%)	
Number of tendons repaired					
	1	67 (72.04%)	37 (45.68%)	104 (59.77%)	
	2	21 (22.58%)	22 (27.16%)	43 (24.71%)	
	3	3 (3.23%)	7 (8.64%)	10 (5.75%)	
	4	2 (2.15%)	4 (4.94%)	6 (3.45%)	
	5	0 (0%)	3 (3.70%)	3 (1.72%)	
	6 or more	0 (0%)	8 (9.87%)	8 (4.59%)	
Concomitant lesion		56 (60.22%)	60 (74.07%)	116 (66.67%)	.053

The 2 groups were largely similar across the studied variables. By design, there was a statistically significant difference between the type of anesthesia performed, with 93 (100%) of patients operated in clinic PRs undergoing a WALANT technique, in contrast to 9 (11.1%) undergoing WALANT in the OR (*P* < .0001). Although not statistically significant, a greater proportion of patients operated in an OR context had concomitant lesions (60 patients, 74.1%), as compared to 56 (60.2%) patients in the clinic-based PR group (*P* = .053).

**Figure 1.**
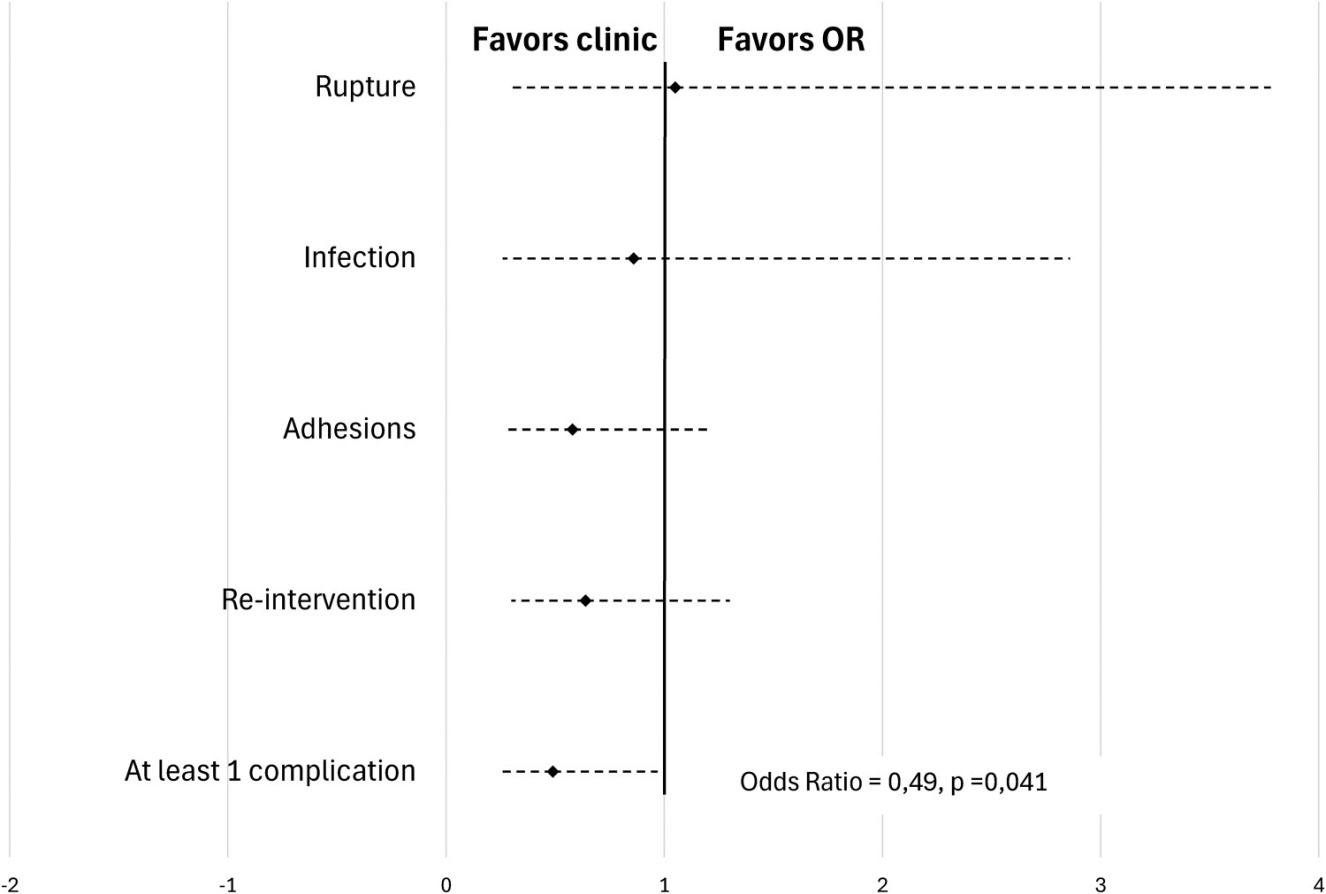
Primary flexor tendon repair rate of complications in single center study population.

No association was observed between surgical setting and presence of postoperative tendon rupture, infectious complications, adhesions and need for a second intervention (see Table 3). A total of 5 ruptures (6.2%) occurred in the OR group and 6 in the PR group (6.5%) (OR = 1.05 [0.30-3.78]; *P* = .94). Infectious complications were noted in 6 cases (7.4%) in the OR group and 6 cases (6.5%) in the PR group (OR = 0.86 [0.26-2.86]; *P* = .800). The presence of adhesions in the postoperative follow-up period was seen in 19 (23.5%) cases in the OR group and 14 cases (15.1%) in the PR group (OR = 0.58 [0.26-1.2], *P* = .160). 19 patients (23.5%) in the OR group required a second intervention, in comparison with 15 patients (15.0%) in the PR group (OR = 0.064 [0.30-1.30]; *P* = .24) (Figure 1). Table 4 summarizes the cases needing reintervention.

**Table 3.** Complications*.

	Outpatient clinic (*n* = 93)	Operating room (*n* = 81)	Total (*n* = 174)	*P*-value
Infection	6 (6.45%)	6 (7.41%)	12 (6.90%)	.80
Tendon rupture	6 (6.45%)	5 (6.17%)	11 (6.32%)	.94
Adhesions	14 (15.05%)	19 (23.46%)	33 (18.97%)	.16
Revision surgery	15 (16.13%)	19 (23.46%)	34 (19.54%)	.24

*Seven patients had 2 complications; 2 patients had all 3 complications.

**Table 4.** Revision Surgeries.

	Outpatient clinic	Operating room	Total
Repair of ruptured tendon	7	6	13
Tenolysis	9	12	21
Washout and debridement of infected wound	3	3	6
Other (ORIF, plate and screw removal, amputation)	1	2	3

In combining the complications (rupture, infection, and adhesions), being operated in a clinic-based PR reduces the chance of complicating by 51% in a statistically significant fashion (OR = 0.49 [0.24-0.97]; *P* = .041). Reintervention was not included in this calculation since it represents more of a proxy for a complication than a true complication in and of itself.

**Figure 2.**
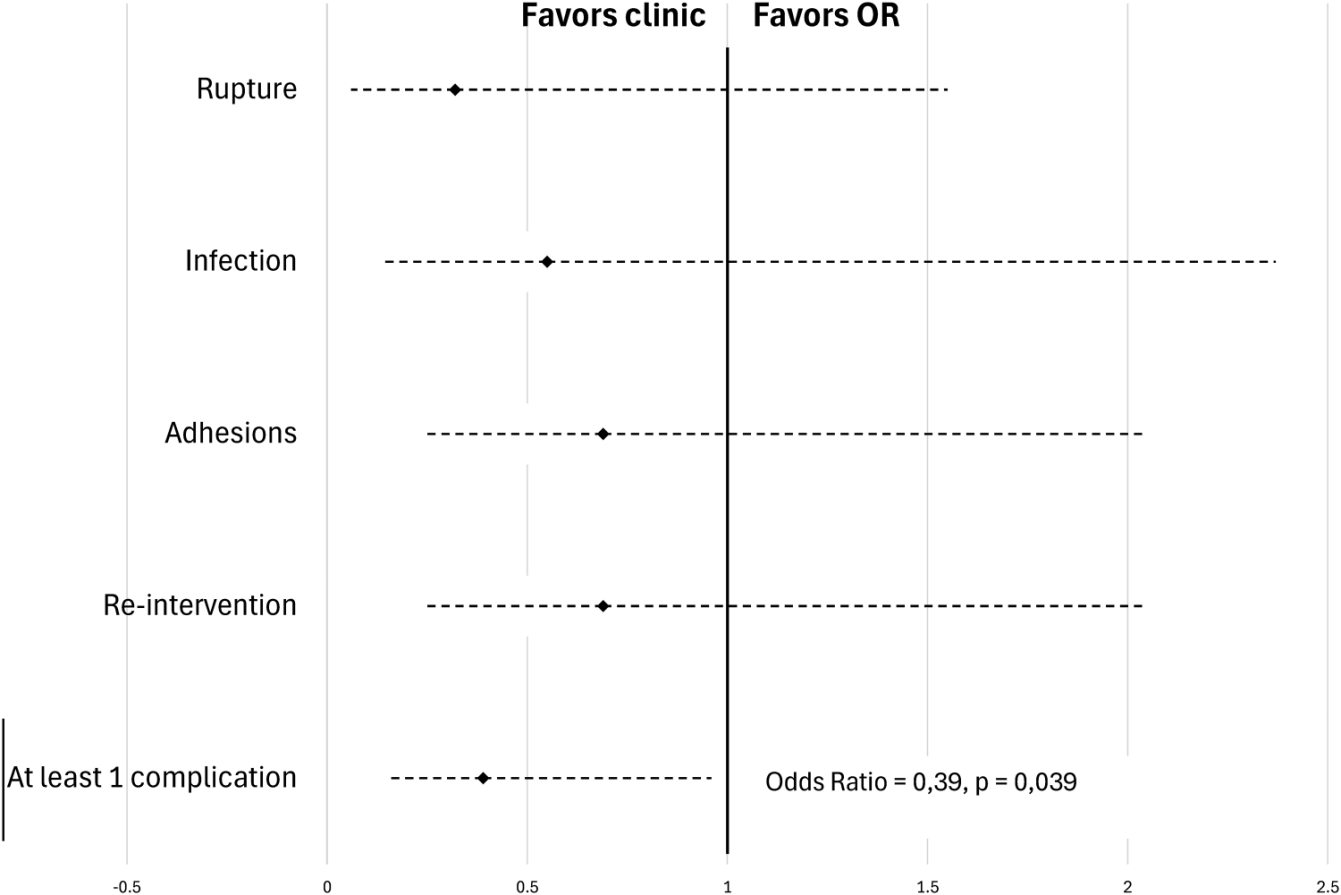
Rate of complications in single-digit subanalysis of flexor tendon repair.

A subgroup analysis was then performed to specifically evaluate patients with injury to one finger alone in the absence of any devascularization injury. No association was observed between surgical setting and presence of rupture (OR = 0.32 [0.06-1.55]; *P* = .154), infection (OR = 0.55 [0.14-2.37]; *P* = .401), adhesion (OR = 0.69 [0.24-2.04]; *P* = .483) or need for reintervention (OR = 0.69 [0.24-2.04]; *P* = .483). In evaluating the presence of at least one complication (rupture, infection, and adhesions), the fact of being operating in an outpatient clinic PR versus OR lead to a statistically significant reduction in the chances of observing a complication by 61% (OR = 0.39 [0.16-0.96]; *P* = .039) (Figure 2).

Table 5 summarizes subgroup analysis by zone of injury. The only statistically significant difference was in zone II, where the PR setting was associated with a 59% reduction in the chance of observing a complication (*P* = .041), and less reintervention was observed as well (32.4% OR vs 11.8% PR; *P* = .049).

**Table 5.** Injury Zone Comparison**.

@		Outpatient clinic	Operating room	*P*-value
Zone I *n* = 58		*n *= 41	*n *= 17	
	Rupture	4 (9.75%)	2 (11.75%)	1.0
	Infection	5 (12.20%)	2 (11.75%)	1.0
	Adhesions	7 (17.07%)	3 (21.43%)	1.0
	Reintervention	9 (21.95%)	2 (11.75%)	.48
	At least 1 complication	9 (21.95%)	6 (35.29%)	.33
Zone II *n* = 71		*n *= 34	*n *= 37	
	Rupture	1 (2.94%)	1 (2.70%)	1.0
	Infection	1 (2.94%)	4 (10.81%)	.36
	Adhesions	5 (14.71%)	11 (29.73%)	.16
	Reintervention	4 (11.76%)	12 (32.43%)	.049
	At least 1 complication	6 (17.65%)	15 (40.54%)	.041
Zone III *n* = 13		*n *= 6	* n *= 7	
	Rupture	0	2 (28.57%)	.46
	Infection	0	0	N/A
	Adhesions	1 (16.67%)	2 (28.57%)	1.0
	Reintervention	1 (16.67%)	3 (42.86%)	.56
	At least 1 complication	1 (16.67%)	3 (42.86%)	.56
Zone V *n *= 28		*n *= 10	*n *= 18	
	Rupture	1 (10%)	0	.36
	Infection	0	0	N/A
	Adhesions	0	3 (16.67%)	.53
	Reintervention	1 (10%)	2 (11.11%)	1.0
	At least 1 complication	1 (10%)	3 (16.67%)	1.0

** Zone IV not analyzed because *n* = 2.

In the OR group, 25 of the 81 patients waited for more than 12 h for their surgery. Those operated in clinic-based PRs were not subjected to operative waiting times.

## Discussion

The primary goal of this study was to evaluate whether performing primary flexor tendon repair in a PR puts patients at a higher risk of complications compared to patients operated in an OR. Our analysis found that the surgical setting of clinic-based PRs did not augment complication rates as compared to traditional main ORs.

Canadian plastic surgeon Dr Don Lalonde introduced and popularized the concept of WALANT for hand surgery.^13,14^ Numerous subsequent studies have testified to the efficacy and safety of WALANT, which is the injection of a local anesthetic agent, lidocaine, ensuring optimal pain control, with the use of epinephrine, a hemostatic agent, permitting good visualization during surgery and removing the need for a tourniquet.^15^ Seeing as the WALANT technique is what allows for flexor tendon repairs to the performed in settings outside of a traditional OR, our investigation contributes to the existing body of the literature describing WALANT specifically for flexor tendon repair.^16,17^ As noted in similar studies, the advantages of WALANT in the setting of primary flexor tendon repair include the ability to evaluate the repair intraoperatively, for factors such as gliding and gapping.^18^

While other papers stratified their study groups based on type of anesthesia,^19–21^ our study uses operative setting as our independent variable. In fact, 9 of the 81 cases performed in the operative room were done with WALANT as the anesthetic technique. Our findings therefore go a step further and demonstrate that the PR setting has advantages in and of itself. For one, performing flexor tendon repair in a setting outside of the OR also allows for a more judicious use of resources, including a considerable reduction of cost to the healthcare system, encompassing which include costs associated to the operating theater, staffing the anesthetist and nursing team, as well as the cost of hospitalization and the examinations and tests required before general anesthesia.^22^ While a cost, profit and efficiency analysis comparing the 2 surgical settings for flexor tendon repair has not been undertaken, a 2011 study showed that performing open carpal tunnel surgery was 4 times more costly in the OR versus a clinic room.^23^ A Pubmed search of the 2 words “WALANT cost” produced 70 publications that support the fact that WALANT is less expensive than traditional sedation/tourniquet surgery.^24^

In theory, air requirements for ORs are greater in order to ensure aseptic conditions, which could imply that fewer air changes could lead to more surgical site infections. A total of 25 air changes per hour is the standard for the OR. Conversely, 6 changes per hour are required for PRs. Our study showed no difference in infection rates between the 2 groups despite the 4 times less air change in clinic-based PRs. The evidence that increased air change rates truly affect infection rates is slim at best.^25^

Our study has several limitations, beginning with its retrospective design. There is a likely selection bias in that patients were not randomized to the different operative settings. Some important outcome factors, such as range of motion and patient-reported outcomes, were not evaluated. Moreover, confounding factors should be acknowledged. For example, it is possible that the reduction shown in the absolute likelihood of complication is attributable to lesser surgical delay rather than the surgical context in and of itself. A 2019 study by Reito et al observed that a surgical delay of 3 to 7 days was associated with an increased risk of complications.^1^

We conducted a single digit analysis to control for the fact that concomitant lesions may have been favored for surgery in the OR. In accordance with the rest of our findings, our single digit analysis demonstrated no significant difference among each of the chosen complication outcomes between the 2 operative settings studied.

To our knowledge, our cohort study is the largest in the literature looking at flexor tendon repairs performed in non-OR PRs. While we were able to observe a decrease in the absolute likelihood of complication of the PR compared to the OR, our study was underpowered to observe significant differences across specific complications (rupture, infection, adhesion, and reoperation). A multicenter randomized study is needed to help investigate this issue more thoroughly.

## Conclusion

Among patients undergoing flexor tendon repair of the hand, the PR setting did not put patients at higher risk of complications when compared to patients operated in an OR. These results reveal an opportunity to recognize the many advantages of opting for non-OR PR settings for hand surgery. Future research should focus on prospective studies in which patients are randomized to operative setting. A cost-benefit analysis and environmental impact study to understand and quantify the opportunity cost of PRs for primary flexor tendon repair would be of benefit.

## References

[1] Reito A Manninen M Karjalainen T . The effect of delay to surgery on major complications after primary flexor tendon repair. J Hand Surg Asian Pac 2019;24(2):161–168. 10.1142/S2424835519500218 31035884

[2] Dy CJ Hernandez-Soria A Ma Y Roberts TR Daluiski A . Complications after flexor tendon repair: a systematic review and meta-analysis. J Hand Surg [Am] 2012;37(3):543–551.e1, ISSN 0363-5023. doi: 10.1016/j.jhsa.2011.11.006. 22317947

[3] Grandizio LC Graham J Klena JC . Current trends in WALANT surgery: a survey of American Society for Surgery of the hand members. J Hand Surg Global Online 2020;2(4):186–190, ISSN 2589-5141. doi: 10.1016/j.jhsg.2020.04.011. PMC8991637 35415507

[4] Rabinowitz J Kelly T Peterson A Angermeier E Kokko K . In-office wide-awake hand surgery versus traditional surgery in the operating room: a comparison of clinical outcomes and healthcare costs at an academic institution. Curr Orthop Pract. 2019;30(5):429–434. doi: 10.1097/BCO.0000000000000785

[5] Baradaran A Meng F Jaberi MM Finlayson R Thibaudeau S . A comparative cost analysis of local anesthesia versus brachial plexus block for complex hand surgery. Hand (N Y). 2023;18(1_suppl):22S–27S. doi: 10.1177/15589447221092061. Epub 2022 Jun 5. PMID: 35658725; PMCID: PMC9896271. 35658725 PMC9896271

[6] Lalonde D Higgins A . Wide awake flexor tendon repair in the finger. Plast Reconstr Surg Glob Open. 2016;4(7):e797. 10.1097/GOX.0000000000000756 PMC4977125 27536476

[7] Lalonde D . Wide awake local anaesthesia no tourniquet technique (WALANT). BMC Proc. 2015;9(Suppl 3):A81. doi: 10.1186/1753-6561-9-S3-A81

[8] Kadhum M Georgiou A Kanapathy M , et al. Operative outcomes for wide awake local anesthesia versus regional and general anesthesia for flexor tendon repair. Hand Surg Rehabil 2022;41(1):125–130. 34700023 10.1016/j.hansur.2021.10.312

[9] El-Gammal TA Saleh WR Ragheb YF Morsy M Ibrahim MA Fekry MS. Outcomes of zone II flexor tendon repair under general versus wide awake local anesthesia: a randomized controlled trial. J Hand Surg Am 2024:49(11):1095–1103. doi: 10.1016/j.jhsa.2024.06.008. PMID: 39115486. 39115486

[10] Søreide K Hallet J Matthews JB , et al. Immediate and long-term impact of the COVID-19 pandemic on delivery of surgical services. Br J Surg. 2020;107(10):1250–1261. doi: 10.1002/bjs.11670. Epub 2020 Apr 30. PMID: 32350857; PMCID: PMC7267363. 32350857 PMC7267363

[11] Billig JI , Sears ED . The compounding access problem for surgical care: innovations in the post-COVID Era. Ann Surg. 2020;272(2):e47–e48. doi: 10.1097/SLA.0000000000004085 PMC7268831 32675492

[12] Cullen S Wrafter PF Jones D , et al. Plastic surgery procedure unit: a streamlined care model for minor and intermediate procedures: a cost-benefit analysis. J Plast Reconstr Aesthet Surg. 2021;74(1):192–198. doi: 10.1016/j.bjps.2020.08.100. Epub 2020 Sep 19. PMID: 33129699. 33129699

[13] Lalonde D . Wide awake local anaesthesia no tourniquet technique (WALANT). In: BMC proceedings. Vol. 9(Suppl 3). BioMed Central, 2015.

[14] Lalonde D Bell M Benoit P Sparkes G Denkler K Chang P . A multicenter prospective study of 3,110 consecutive cases of elective epinephrine use in the fingers and hand: the Dalhousie project clinical phase. J Hand Surg [Am]. 2005;30(5):1061–1067. doi: 10.1016/j.jhsa.2005.05.006 16182068

[15] Thomson CJ Lalonde DH Denkler KA , et al. A critical look at the evidence for and against elective epinephrine use in the finger. Plast Reconstr Surg. 2007;119(1):260–266. 17255681 10.1097/01.prs.0000237039.71227.11

[16] Elhameed A Adel M . The outcome of the WALANT technique in primary hand flexor tendons repair. JPRAS Open 2024;40:77–84. 38444624 10.1016/j.jpra.2023.11.012 PMC10914414

[17] Kadhum M Georgiou A Kanapathy M , et al. Operative outcomes for wide awake local anesthesia versus regional and general anesthesia for flexor tendon repair. Hand Surg Rehabi 2022;41(1):125–130. 10.1016/j.hansur.2021.10.312 34700023

[18] Lalonde DH Sepehripour S . Tips to successful flexor tendon repair and reconstruction with WALANT. Hand Clin. 2023;39(2):165–170. doi: 10.1016/j.hcl.2022.08.017. Epub 2023 Feb 14. PMID: 37080648. 37080648

[19] Bamal R Alnobani O Bastouros E , et al. Wide-awake local anesthesia no tourniquet (WALANT) for flexor tendon repairs as change in practice during the COVID-19 pandemic: a retrospective cohort study with outcomes. Cureus. 2023;15(3):e36728. doi: 10.7759/cureus.36728. PMID: 37123769; PMCID: PMC10131134. PMC10131134 37123769

[20] El-Gammal TA Saleh WR Ragheb YF Morsy M Ibrahim MA Fekry MS . Outcomes of zone II flexor tendon repair under general versus wide awake local anesthesia: a randomized controlled trial. J Hand Surg Am 2024;49(11):1095–1103. doi: 10.1016/j.jhsa.2024.06.008. PMID: 39115486. 39115486

[21] Elhameed MAA Hassan KM Metawally AMA Sabry M . The outcome of the WALANT technique in primary hand flexor tendons repair. JPRAS Open. 2023;40:77–84. doi: 10.1016/j.jpra.2023.11.012. PMID: 38444624; PMCID: PMC10914414. 38444624 PMC10914414

[22] Pires PJ de A Moreira L de Las Casas PP . É seguro o uso de anestésico local com adrenalina na cirurgia da mão? Técnica WALANT⋆. Rev Bras Ortop 2017;52(4):383–389. doi:10.1016/j.rbo.2017.05.002 28884094 PMC5582825

[23] Chatterjee A McCarthy JE Montagne SA et al. A cost, profit, and efficiency analysis of performing carpal tunnel surgery in the operating room versus the clinic setting in the United States. Ann Plast Surg. 2011;66(3): 245–248. 21042185 10.1097/SAP.0b013e3181db7784

[24] WALANT cost - Search Results. PubMed (nih.gov) 70 publications produced with a Pubmed search accessed on Aug 24, 2024.

[25] Silver N Lalonde DH . Main operating room versus field sterility in hand surgery: a review of the evidence. Plast Surg. 2023;32(4):627–637. doi: 10.1177/22925503231161073 PMC11492193 39439664

